# Forecasting the potential impact of cell and gene therapies in France: projecting product launches and patients treated

**DOI:** 10.3389/fmed.2024.1324602

**Published:** 2024-02-19

**Authors:** Ming Kei Lee, Sama Seyedmousavi, Sylvain Auvity, Bertrand Pourroy, Vincent Elleboode, Isabelle Kachaner, Christel Jansen, Herve Lilliu

**Affiliations:** ^1^Inbeeo, London, United Kingdom; ^2^Université Paris Cité, INSERM, UMRS-1144, Optimisation Thérapeutique en Neuropsychopharmacologie, Paris, France; ^3^Service de Pharmacie, AP-HP, Hôpital Necker, Paris, France; ^4^Oncopharma Unit, Pharmacy Department, University Teaching Hospital la Timone, Marseille, France; ^5^Pfizer Holding France, Paris, France

**Keywords:** cell and gene therapies, patient forecast model, pharmaceutical development pipeline, French healthcare system, patient access, healthcare organization and delivery

## Abstract

**Objective:**

To evaluate the potential impact of cell and gene therapies (CGTs) in France by forecasting the number of patients that will be treated with CGTs over the period 2023–2030 by therapeutic area and region.

**Methods:**

A review of CGTs in clinical development and related disease epidemiology was conducted to forecast the number of CGT launches and patient population between 2023 and 2030. The number of expected launches was identified by filtering the clinical development pipeline with estimated time to launch and probability of success values from Project ALPHA. Disease prevalence and incidence in France were combined with projected adoption rates derived from historical data to forecast the patient population to be treated.

**Results:**

Up to 44 new CGTs are forecasted to launch in France in the period 2023–2030, which translates into more than 69,400 newly treated patients in 2030. Leading indications in terms of newly treated patients per year include cardiovascular disease, hematological cancers and solid tumors with 27,300, 15,200 and 13,000 newly treated patients in 2030, respectively.

**Discussion:**

The forecast suggests that the future landscape of CGTs will undergo a shift, moving from CGTs targeting (ultra) rare diseases to more prevalent diseases. In France, this will likely pose organizational challenges hindering patient access to these transformative therapies. Further research and planning around network organization and patient distribution are needed to assess and improve the readiness of the French healthcare system for ensuring access for this growing number of patients to be treated with CGTs.

## 1 Introduction

As of 31 December 2022, 13 cell and gene therapies (CGTs), defined as gene therapy medicinal products and somatic cell therapies, excluding tissue-engineered therapies, were granted a European marketing authorization (1, 2). Out of these, 10 have been reimbursed or received early access authorization (AAP) in France, as shown in Table 1.

**Table 1:** List of authorized CGTs and their availability in France, as per 31 Dec 2022 (2).

**Generic name**	**Brand name**	**Therapy type**	**Indication**	**Reimbursement status in France**
Tisagenlecleucel	Kymriah	*Ex vivo*	DLBCL, ALL, FL	Reimbursed, AAP for FL
Axicabtagene ciloleucel	Yescarta	*Ex vivo*	DLBCL, FL	Reimbursed, AAP for FL
Brexucabtagene autoleucel	Tecartus	*Ex vivo*	MCL	Reimbursed
Voretigene neparvovec	Luxturna	*In vivo*	Inherited retinal dystrophy	Reimbursed
Onasemnogene abeparvovec	Zolgensma	*In vivo*	SMA	AAP
Atidarsagene autotemcel	Libmeldy	*Ex vivo*	MLD	AAP
Idecabtagene vicleucel	Abecma	*Ex vivo*	MM	AAP
Lisocabtagene maraleucel	Breyanzi	*Ex vivo*	DLBCL, PMBCL	AAP
Ciltacabtagene autoleucel	Carvykti	*Ex vivo*	MM	AAP
Eladocagene exuparvovec	Upstaza	*In vivo*	AADC	AAP
Talimogene laherparepvec	Imlygic	*In vivo*	Melanoma	Not assessed
Autologous CD34+ enriched cell fraction	Strimvelis	*Ex vivo*	ADA-SCID	Not assessed
Valoctocogene roxaparvovec	Roctavian	*In vivo*	Hemophilia A	HTA assessment ongoing

AADC, aromatic L-amino acid decarboxylase; AAP, early access authorization (autorisation d'accès précoce); ADA-SCID, Adenosine deaminase deficiency severe combined immunodeficiency; ALL, Acute lymphoblastic leukemia; DLBCL, Diffuse large B cell lymphoma; FL, Follicular lymphoma; MCL, Mantle cell lymphoma; MLD, metachromatic leukodystrophy; MM, multiple myeloma; PMBCL, primary mediastinal large B-cell lymphoma; SMA, Spinal muscular atrophy.

With over 2,000 potential CGTs in pipeline development (3–7), it is imperative to evaluate the impact of these disruptive therapies on health systems for three reasons.

Firstly, CGTs have the potential to offer important and potentially curative health benefits to patients, sometimes following a one-time administration instead of chronic treatment (8). Chimeric antigen receptor T-cell therapies such as Tisagenlecleucel (Kymriah^®^) and Axicabtagene ciloleucel (Yescarta^®^) have demonstrated significant clinical outcomes in treating B-cell malignant diseases (9). A 5-year data report on Kymriah showed a 55% overall survival rate, with a median event-free survival period of 43.8 months for the 82% of acute lymphoblastic leukemia patients who experienced remission within 3 months of infusion (10). Moreover, results from a 5-year follow-up study for Yescarta^®^ demonstrated a 42.6% overall survival rate of the B cell lymphoma patients who received a single infusion in the period and 40.5% of patients who experienced remission at 2 years (11, 12). Another example would be Onasemnogene abeparvovec (Zolgensma^®^) for patients with spinal muscular atrophy. Recent long-term data showed all patients in the presymptomatic intravenous cohort maintained or achieved all assessed motor milestones (13).

Secondly, these modalities often come with high upfront price tags. This is mainly due to the high costs of goods and the fact that CGTs typically deliver long-term value following a one-off treatment (8, 14, 15). For reimbursed CGTs, the list price ranges between €290,000 to 360,000 (16, 17). For CGTs under AAP which includes Zolgensma^®^, Libmeldy^®^ and Upstaza^®^, list prices range between €1,945,000 to 3,500,000 (18, 19). As more CGTs enter the market in the future, the high upfront prices may challenge the sustainability and affordability of the existing funding system, as demand for new innovative CGTs might exceed allocated healthcare budgets (20, 21).

Lastly, forthcoming CGTs might also pose challenges for healthcare service delivery. At the moment, most CGTs are targeting orphan or ultra-rare diseases where treatments are facilitated in a limited number of specialist centers (5). Considering the vast array of potential CGTs on the horizon (6, 22), hospitals might get overwhelmed to accommodate the respective patients due to various factors including a lack of qualified treatment centers and scarcity of expert personnel (23, 24).

The objective of this study is to evaluate the impact of CGTs in France by forecasting the number of patients that will be treated with CGTs over the period 2023–2030 in the different therapy areas and regions.

## 2 Methods

**Figure 1:**
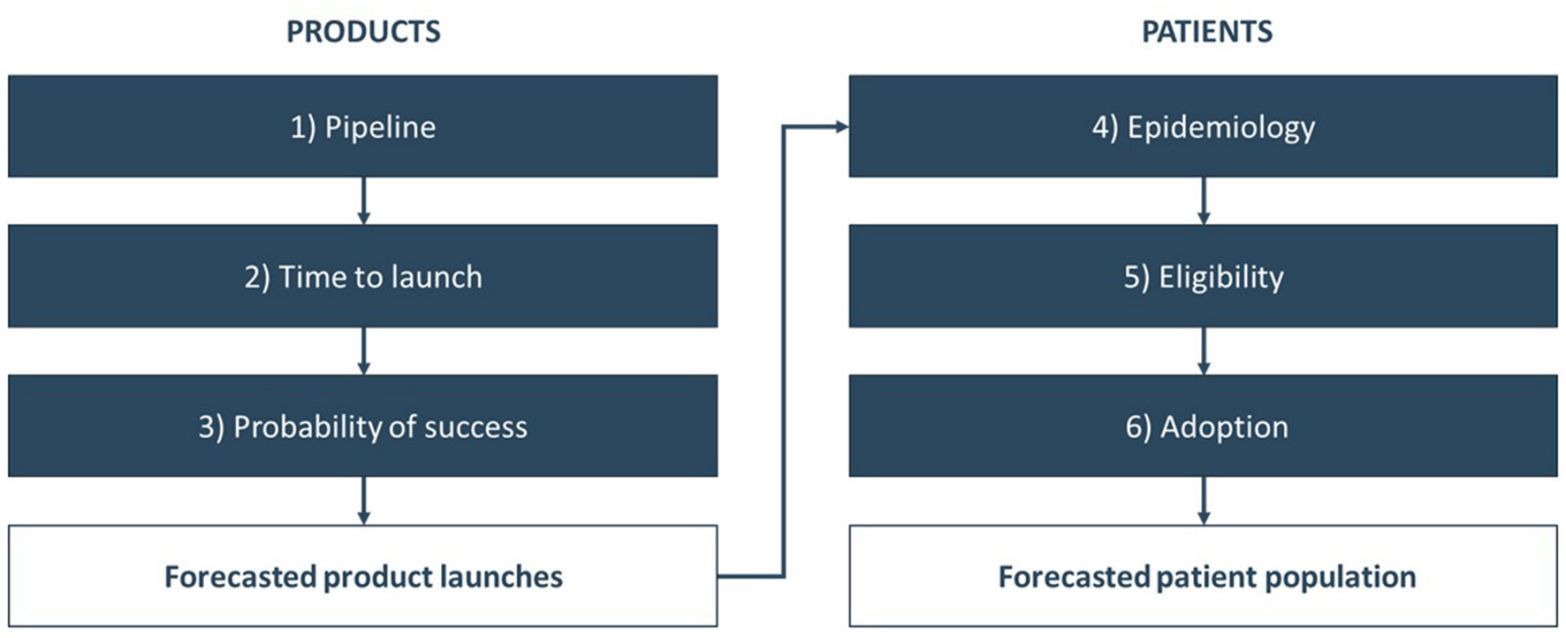
Schematic overview of the patient-based, epidemiological forecast of CGTs launches and use.

To gain a more accurate understanding of the upcoming wave of CGTs and their forecasted use, we adopted a patient-based, epidemiological approach to forecast the patient population. A schematic overview of this approach is depicted in Figure 1.

### 2.1 Mapping the CGT clinical development pipeline

Existing CGT clinical trials active in the US and the EU were identified through *clinicaltrails.gov* (25) and *Catapult.org.uk* (26), using the following search terms, in line with the definition of CGTs used in this study: gene correction, CRISPR/CAS9 gene editing, Chimeric Antigen T- cells therapies, and T cell receptor. Trials with therapies that did not involve gene modification (e.g., human stem cell transplantation without genetic modification, or other forms of cellular transplantation) were excluded. Basket trials covering the application of one CGT in multiple indications were separated and assessed independently.

The product profile, indication, therapy area, development stage (Phase I, Phase II or Phase III), trial status (active, recruiting, not yet recruiting, or enrolling by invitation) and estimated and actual study completion dates were extracted for each of the trials adhering to our search criteria. Data extraction covered all the trial information available in December 2022.

### 2.2 Forecasting time to launch

Timing to first patient was defined as the number of years required to reach commercial launch in France from an asset's current clinical phase. This forecast was based on the average trial duration of 28 FDA-approved and 25 EMA-approved CGTs (1, 27). Given their relevance for France, EMA approved data points were used in the case of approval by both FDA and EMA. To account for inherent uncertainty, three launch scenarios “Pessimistic launch,” “Base launch” and “Optimistic launch” were created. The main results presented here were based on the “Base launch” scenario, in which the assumed duration from Phase I, Phase II and Phase III to launch were 9, 7, and 5 years, respectively. Based on an analysis of the difference between the estimated and actual date of completion for each product in the CGT pipeline mapped in step 1, the duration for the pessimistic and optimistic launch scenarios has a 1-year difference from the base launch. Subsequently, the forecasted product launches were identified on a year-by-year basis, until 2030. The timing of early access programs was not considered in the analysis.

### 2.3 Forecasting the probability of success

The probability of success (PoS) for a CGT obtaining marketing authorization was estimated based on its clinical development phase, using data from the Massachusetts Institute of Technology project ALPHA (Analytics for Life-sciences Professionals and Healthcare Advocates) (28, 29). Project ALPHA uses historical data from completed clinical trials globally to provide the likelihood of success for each phase of a clinical trial. The PoS were used to forecast the success or failure of each asset. Table 2 below shows the PoS for each phase.

**Table 2:** CGTs Probability of Success estimation based on MIT's project ALPHA.

**Phase of development**	**Probability of success**
Phase 1	10.8%
Phase 2	16.0%
Phase 3	44.1%

### 2.4 Determining incidence and prevalence rate per indication

The incidence and prevalence of forecasted CGTs' indication were extracted from various online sources:

- Orphanet (30) and Nord (31) for rare and ultra-rare diseases
- The French National Cancer Institute (32), Global Cancer Observatory (33), and Cancer Research UK (34) for oncology
- Literature publications and French national reports for non-rare and non-oncology diseases.

In case of discrepancies between databases, the lowest available prevalence and/or incidence estimates were used. The prevalence and incidence rates of expected CGT indications were applied to the 2022 French population to determine the eligible patient population size on a year-by-year basis (35, 36).

### 2.5 Estimating treatment eligibility per CGT

A product might ultimately be used for a specific subpopulation instead of the full indication for which it was initially developed. Subpopulations can be defined based on various criteria such as particular genetic mutations, disease severity, age, or line of treatment (37). To forecast the patient population eligible for receiving a CGT in development, the target patient population for each CGT was estimated by examining the inclusion and exclusion criteria listed in trial designs and reviewing the reimbursement outcomes of marketed CGTs.

### 2.6 Forecasting the adoption rate

Adoption rates within the subpopulation were estimated using historical data of approved products where the number of treated patients in a year was compared to the eligible patient population (38, 39). Based on the analysis looking at 11 approved CGTs, the adoption rates ranged from 1 to 30%. Given that most forecasted CGTs will target oncology indications as suggested in results, this led to an estimated 1% adoption rate for each product in year one of launch, with incremental increases of 1% per year to a maximum of 6% in year six. In the case where multiple CGTs will be available in the same indication, the total eligible population per indication was divided by the number of CGTs.

## 3 Results

The mapping of the CGT clinical development pipeline led to the identification of 749 individual clinical trial programs for 419 CGTs in 38 indications. The clinical trial programs comprised 327 phase I studies, 367 phase II studies, and 55 phase III studies. A breakdown of these is shown in Supplementary Table 2.

**Figure 2:**
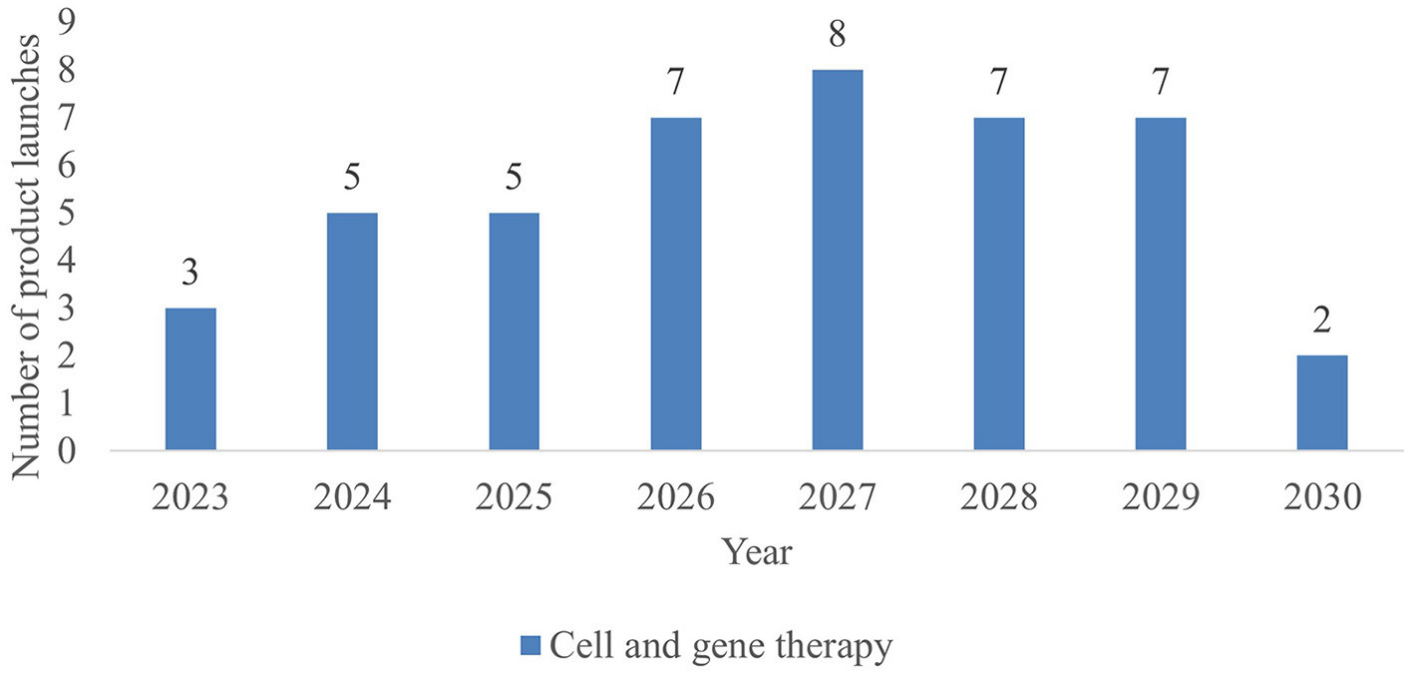
Forecasted CGT launches per year in France, 2023–2030.

As shown in Figure 2, 44 new CGTs are expected to reach the French market by 2030. An increase in CGT launches is forecasted from 2023 to 2027, followed by a decline after 2027. Since most products to be launched post 2027 are currently in phase I development resulting in a lower PoS value, this number is likely to increase along with the progression of CGTs in the clinical development pipeline. Forecasted CGT launches across three scenarios are shown in the Supplementary Figure 1.

During 2023–2030, more than half (57%) of launched CGTs are targeting non-oncological diseases, predominately metabolic (such as Aromatic L-Amino Acid Decarboxylase Deficiency and Fabry Disease) and ophthalmological diseases (such as inherited retinal dystrophy and dry age-related macular degeneration). A breakdown of CGTs by therapeutic area is shown in Table 3. A list of CGT's target indications is shown in Supplementary Table 3.

**Table 3:** Forecasted CGT most expected launches per year in France by therapy area, 2023–2030.

**Therapy area**	**2023**	**2024**	**2025**	**2026**	**2027**	**2028**	**2029**	**2030**	**Total**
Haemato-Oncology	1	0	0	1	2	3	3	1	11
Solid tumor	0	1	0	0	3	1	3	0	8
Metabolic	0	1	2	2	0	1	0	1	7
Ophthalmology	0	2	0	2	0	2	0	0	6
Hematology (non-oncology)	2	0	0	0	1	0	1	0	4
Neurology	0	1	2	1	0	0	0	0	4
Immunology	0	0	0	0	2	0	0	0	2
Cardiovascular	0	0	1	0	0	0	0	0	1
Dermatology	0	0	0	1	0	0	0	0	1
Total	3	5	5	7	8	7	7	2	

**Figure 3:**
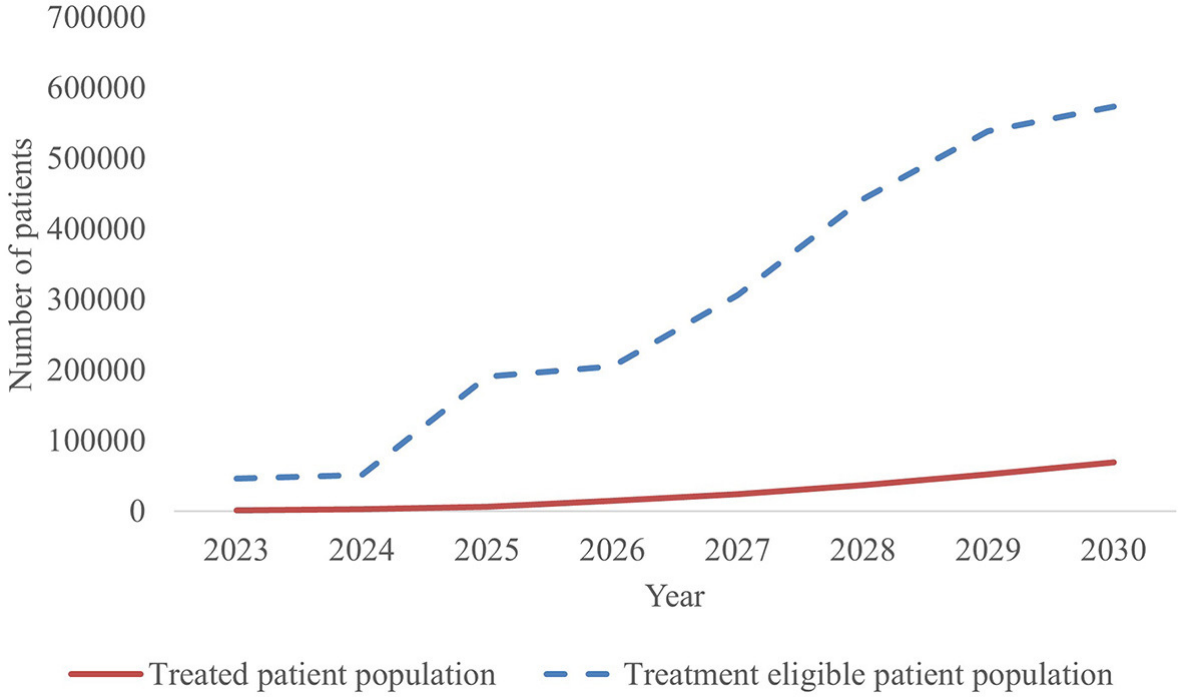
Forecasted population of newly treated patients per year in France, 2023–2030.

With 44 new CGTs the most expected to reach the French market, the projected annual number of treated patients increases from 1,653 in 2023 to more than 69,400 patients by 2030, as shown in Figure 3.

**Figure 4:**
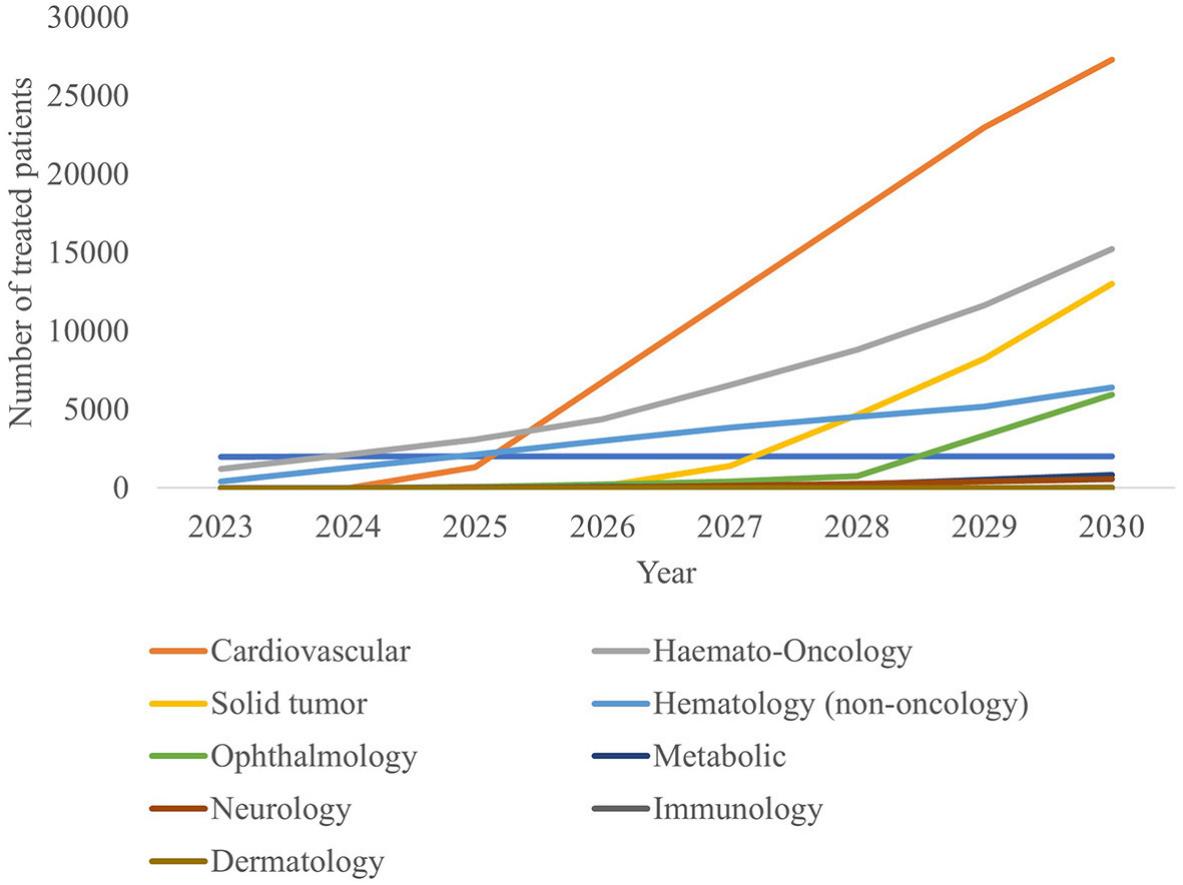
Number of annual treated patients in France by therapy area, 2023-2030.

The estimated number of newly treated patients per year is broken down by therapy area in Figure 4 and Table 4. By 2030, leading disease groups based on the annual number of newly treated patients include cardiovascular diseases, hematological cancers, and solid tumors with respectively 27,321, 15,217 and 13,030 newly treated patients per year. For hematological cancers, this number is expected to increase at a steady rate from 2023, whereas the population sizes of cardiovascular diseases and solid tumor cancers are expected to escalate beyond 2025 and 2027, respectively.

**Table 4:** Number of annual treated patients in France by therapy area, 2023–2030.

**Disease group**	**2023**	**2024**	**2025**	**2026**	**2027**	**2028**	**2029**	**2030**	**Total**
Cardiovascular	0	0	1,353	6,763	1,2173	17,583	22,993	27,321	88,185
Haemato-Oncology	1,224	2,153	3,082	4,376	6,556	8,820	11,646	15,217	53,075
Solid tumor	0	14	68	122	1,420	4,656	8,237	13,030	27,546
Hematology (non-oncology)	429	1,286	2,144	3,001	3,859	4,558	5,192	6,428	26,896
Ophthalmology	0	7	88	237	426	751	3,368	5,951	10,827
Metabolic	0	1	14	58	98	211	534	844	1,761
Neurology	0	2	19	51	159	267	410	576	1,484
Immunology	0	0	0	0	4	9	15	20	49
Dermatology	0	0	0	1	4	7	9	12	34
Total	1,653	3,463	6,768	14,609	24,699	36,862	52,404	69,400	

## 4 Discussion

### 4.1 By 2030, an increasing number of CGTs in increasingly prevalent indications is to be expected

Model outputs forecast up to 44 CGTs to reach the French market in the period 2023–2030. This translates into more than 67,000 newly treated patients in 2030. Leading indications in terms of newly treated patients per year include cardiovascular disease, hematological cancers and solid tumors with ~27,300, 15,200 and 13,000 newly treated patients in 2030, respectively.

The forecasted product launches and patient population suggest that the future landscape of CGTs will undergo a shift moving from targeting (ultra) rare diseases to more prevalent diseases (Table 4). Currently, available CGTs mostly target (ultra) rare diseases which often have a genetic origin. The shift to more prevalent diseases of genetic origin might lead to a larger patient population than previously anticipated (40, 41).

### 4.2 Model limitations to consider when interpreting the findings

Several characteristics of the forecasting model need to be considered when interpreting the forecasting results.

First of all, the CGT development space is very dynamic with new clinical studies commencing on a regular basis. Yet, this forecast of the CGT impact is based on a snapshot of the clinical pipeline in December 2022. Subsequent changes to the clinical development pipeline are not considered. As market entry of new CGTs often requires ten-plus years (40), the forecasting time horizon was narrowed to 2023–2030 to reduce the impact of clinical pipeline changes to a minimum.

Secondly, the PoS assumptions sourced from project ALPHA are mainly based on small molecule products, which might differ from the PoS for CGT products given their difference in R&D and clinical development process (28, 29). With only 13 CGTs authorized in Europe to provide historical data on the PoS, the project ALPHA database was considered the most reliable source of information for this analysis.

Thirdly, not accounting for the regulatory or reimbursement process time in estimating the timing to launch can also be seen as a limitation, as reimbursement timelines in France may take 653 days on average in the case of orphan medicines (41). However, these processes for CGTs are often shortened with early access programs such as accelerated approval and early access authorization (AAP) (42). These programs were predominantly developed to expedite direct access to therapies for indications with significant unmet medical needs, particularly for patients lacking variable therapeutic alternatives. Due to the potential curative characteristics of these therapies, a number of approved CGTs made available through the AAP scheme in France. Yet, it remains uncertain whether, forthcoming CGTs targeting more prevalent indications will also be accessible through the AAP program. Moreover, there is a valid concern regarding the sustainability of this scheme in accommodating the anticipated influx of upcoming therapies.

Lastly, a major element driving the forecasted number of newly treated patients per year is the adoption rate. The assumed adoption rate of 1% in the first year after launch to a maximum of 6% in year six post-launch was applied across CGTs. In reality, adoption rates are likely to vary depending on the unmet need and level of competition within the indication (40, 43, 44).

Despite these limitations, the population of patients eligible for CGTs is growing rapidly, which is likely to pose challenges which could hinder patient access to these therapies in France.

### 4.3 The rapidly growing number of patients eligible for CGTs is likely to pose organizational challenges in France

Within the hospital, pharmacy units are largely responsible for managing CGTs, from ordering to patient delivery. Compared to small molecules or classical biologic medicines, managing CGTs requires additional staffing, expertise, equipment, space, and processes for hospital pharmacies.

First, reception of the CGT delivery requires at least two dedicated staff members for at least 30 min. This differs from traditional medicines, where a single staff member can handle multiple deliveries. Second, specific and expensive equipment is needed, among others for cold and ultra-cold storage means and CGT manipulation. Third, CGT preparation requires dedicated space and up to 4 h of work by 2 or 3 pharmacists. Fourth, CGT infusion requires nursing support for longer infusion times and post-administration observation, especially with the arrival of mRNA-based technologies which require multiple and repeated injections instead of a single injection regimen. And lastly, processes, time, and expertise (under the auspices of a specialty pharmacist and with technical support from manufacturers) need to be put in place to ensure:

- Smooth coordination and transportation between the pharmacy and the clinical units, especially in case of cold-chain transportation and low stability durations.
- Efficient delivery to patients without spillage, given the financial risk involved.
- Appropriate handling of liquid nitrogen, genetically modified organisms and related waste.
- Regular equipment monitoring.
- A robust and traceable staff training system to support the new responsibilities.

Yet, so far, hospital pharmacies in France have implemented CGTs with no additional and/or dedicated staffing (45). Maintaining the status quo will significantly limit the number of patients that can be treated per day or week, leading to delays in CGT treatment and difficult decisions regarding patient prioritization. To ensure patient access to these transformative therapies, French stakeholders will have to strengthen existing centers in terms of staffing, expertise, equipment, space, and processes for hospital pharmacies, through targeted funding.

### 4.4 Planning around network organization and patient distribution is needed to ensure patient access to these transformative therapies

Apart from enhancing the capacity of existing centers, it is also important to expand the CGT infrastructure by increasing the number and optimizing the distribution of centers across France. In 2022, a comparison analysis of the number and distribution of specialist treatment centers was conducted across key markets (19). In France, there were 0.046 treatment centers per capita (per 100,000 population) and 0.0543 centers per 1,000 square km. Although the number of centers per capita in France was higher than the EU average (0.028 per capita), the geographic density of centers was lower than most EU countries (0.071 per square km). This suggests potential access challenges for patients living at a great distance from available centers and therefore highlights the importance of expanding infrastructure coverage to ensure patient access.

### 4.5 The regulatory environment for CGTs is changing, which may impact future launches

The proposed revision of the EU pharma legislation might influence future CGT launches, as these revisions include a potential change in incentives for products targeting a high unmet need or rare disease. However, more published information and implementation details will be needed to understand the magnitude of impact (46).

Furthermore, in response to the budgetary impact challenges of CGTs on the healthcare system, the French authorities will enforce outcome-based agreements with installment payments as part of Article 54 of the Social Security Finance Act 2023 (47). The terms of these agreements, including the number of payments, the level of payments, conditions and timelines will be negotiated between the manufacturer and the Comité Économique des Produits de Santé (CEPS) (48, 49). This legislation could improve access to CGTs in France by addressing the financial risks associated with these therapies (50), yet will also bring new organizational challenges related to data infrastructure to measure CGT outcomes.

## 5 Conclusion

France will face an incoming wave of new CGTs in the coming years which means a surge in the patient population that will challenge the current healthcare system. While this study provides an estimation regarding the magnitude of future CGT impact and outlines the potential challenges France may face, the identified limitations underscore the need for ongoing research. Further research and planning are needed to assess and improve the readiness of the French healthcare system to ensure access for this growing number of patients to be treated with CGTs. This includes:

- Establishing a reference network of specialized and accessible CGT treatment centers to accommodate eligible CGT patients.
- Planning for development of clear CGT handling processes, and expansion of required infrastructure, equipment, and qualified staff in GCT treatment centers.
- Organizing the data infrastructure for outcome-based agreements.
- Enhancing the understanding of the financial impact and potential cost savings of these disruptive therapies.

## Data availability statement

The original contributions presented in the study are included in the article/Supplementary material, further inquiries can be directed to the corresponding author.

## Author contributions

ML: Conceptualization, Formal Analysis, Investigation, Methodology, Project administration, Supervision, Validation, Writing—original draft, Writing—review & editing, Data curation, Visualization. SS: Formal Analysis, Investigation, Methodology, Writing—original draft, Writing—review & editing, Data curation, Visualization. SA: Supervision, Validation, Writing—original draft, Writing—review & editing. BP: Supervision, Validation, Writing—original draft, Writing—review & editing. VE: Conceptualization, Supervision, Validation, Writing—review & editing, Methodology, Resources. IK: Supervision, Validation, Writing—review & editing, Conceptualization, Resources. CJ: Conceptualization, Project administration, Supervision, Validation, Writing—original draft, Writing—review & editing. HL: Conceptualization, Project administration, Supervision, Validation, Writing—review & editing, Data curation, Funding acquisition, Methodology.
