# Supplementary material for: Forecasting the potential impact of cell and gene therapies in France: projecting product launches and patients treated
Source: Front Med (Lausanne). 2024 Feb 19;11:1324602. doi: 10.3389/fmed.2024.1324602 (PMC10910012; doi:10.3389/fmed.2024.1324602)
Supplement: Supplementary file 1 [file Table_1.DOCX]

**Table S1**. Estimated timing from Phase I, II and III to launch for three created scenarios.

| Development stage | Optimistic launch | Base launch | Pessimistic launch |
| --- | --- | --- | --- |
| Phase I | 8 years | 9 years | 10 years |
| Phase II | 6 years | 7 years | 8 years |
| Phase III | 4 years | 5 years | 6 years |

**Table S2**. Overview of clinical trials for CGTs across different diseases.

| Disease group | Phase I | Phase II | Phase III | Total |
| --- | --- | --- | --- | --- |
| Cardiovascular | 7 | 16 | 4 | 27 |
| Dermatology | 0 | 2 | 2 | 4 |
| Haematology (non-oncology) | 13 | 30 | 8 | 51 |
| Haemato-Oncology | 103 | 51 | 6 | 160 |
| Immunology | 6 | 24 | 0 | 30 |
| Metabolic | 23 | 69 | 9 | 101 |
| Neurology | 30 | 40 | 10 | 80 |
| Solid tumour | 108 | 71 | 1 | 180 |
| Ophthalmology | 14 | 48 | 14 | 76 |
| Total | 304 | 351 | 54 |  |

Note: Data extraction covered all the trial information available as of January 2023

**Table S3**. List of forecasted CGT’s target indication in France, 2023-2030.

| Forecasted year of launch | Forecasted CGTs indication |
| --- | --- |
| 2023 | - Haemophilia B - Sickle Cell Disease - Mantle cell lymphoma and acute lymphoblastic leukemia |
| 2024 | - Choroideremia - Aromatic L-amino Acid Decarboxylase (AADC) Deficiency - Recurrent Glioblastoma Multiforme - Limb Girdle Muscular Dystrophies (LGMD) - Amaurosis |
| 2025 | - Homozygous Familial Hypercholesterolemia (HoFH) - Variant Late-Infantile Neuronal Ceroid Lipofuscinosis - Peripheral Arterial Disease - Spinal Muscular Atrophy - X-Linked Chronic Granulomatous Disease |
| 2026 | - Ornithine Transcarbamylase (OTC) Deficiency - Leber's hereditary optic neuropathy - Epidermolysis Bullosa - Hodgkin Lymphoma - Achromatopsia - Severe Combined Immunodeficiency, X-Linked - Hemophilia B |
| 2027 | - Lymphoma - Neoplasms - Glioblastoma Multiforme - Pancreatic Cancer - ADA-SCID - Lysosomal Storage Disease - Duchenne Muscular Dystrophy - B-Cell Non Hodkin Lymphoma |
| 2028 | - X-Linked Retinitis Pigmentosa - Multiple Myeloma - Hemophilia A - HIV-1-related high-risk lymphoma - Fabry Disease - Non Small Cell Lung Cancer - Acute Lymphoblastic Leukemia |
| 2029 | - Dry Age-related Macular Degeneration - Sickle Cell Disease - Chronic Lymphocytic Leukemia - Acute Myeloid Leukemia - Lymphocytes - Hepatocellular Carcinoma - Stage IV Cutaneous Melanoma - Melanoma |
| 2030 | - Acute Lymphoblastic Leukemia - Wilson Disease |
