# Supplementary figures and images for: Forecasting the potential impact of cell and gene therapies in France: projecting product launches and patients treated
Source: Front Med (Lausanne). 2024 Feb 19;11:1324602. doi: 10.3389/fmed.2024.1324602 (PMC10910012; doi:10.3389/fmed.2024.1324602)

**Figure S1.** Forecasted CGT launches across three scenarios per year in France, 2023-2030.

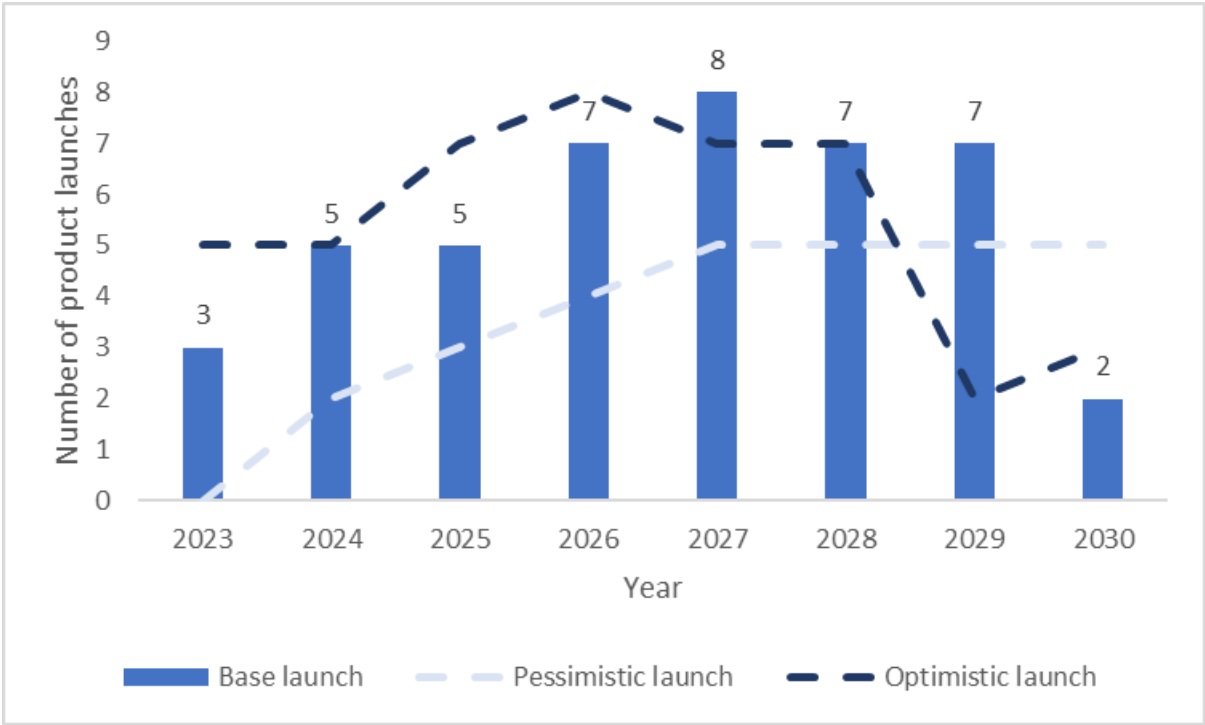

Supplement: Supplementary file 2 [file Data_Sheet_1.PDF]
